# Serum Circulating microRNA Profiling for Identification of Potential Type 2 Diabetes and Obesity Biomarkers

**DOI:** 10.1371/journal.pone.0077251

**Published:** 2013-10-15

**Authors:** Nuria Pescador, Milagros Pérez-Barba, José María Ibarra, Arturo Corbatón, María Teresa Martínez-Larrad, Manuel Serrano-Ríos

**Affiliations:** 1 Spanish Biomedical Research Centre in Diabetes and Associated Metabolic Disorders (CIBERDEM), Madrid, Spain; 2 Instituto de Investigación Sanitaria del Hospital Clínico San Carlos (IdISSC), Madrid, Spain; Central China Normal University, China

## Abstract

**Background and Aim:**

MicroRNAs are small non-coding RNAs that play important regulatory roles in a variety of biological processes, including complex metabolic processes, such as energy and lipid metabolism, which have been studied in the context of diabetes and obesity. Some particular microRNAs have recently been demonstrated to abundantly and stably exist in serum and to be potentially disease-specific. The aim of this profiling study was to characterize the expression of miRNA in serum samples of obese, nonobese diabetic and obese diabetic individuals to determine whether miRNA expression was deregulated in these serum samples and to identify whether any observed deregulation was specific to either obesity or diabetes or obesity with diabetes.

**Patients and Methods:**

Thirteen patients with type 2 diabetes, 20 obese patients, 16 obese patients with type 2 diabetes and 20 healthy controls were selected for this study. MiRNA PCR panels were employed to screen serum levels of 739 miRNAs in pooled samples from these four groups. We compared the levels of circulating miRNAs between serum pools of each group. Individual validation of the twelve microRNAs selected as promising biomarkers was carried out using RT-qPCR.

**Results:**

Three serum microRNAs, miR-138, miR-15b and miR-376a, were found to have potential as predictive biomarkers in obesity. Use of miR-138 or miR-376a provides a powerful predictive tool for distinguishing obese patients from normal healthy controls, diabetic patients, and obese diabetic patients. In addition, the combination of miR-503 and miR-138 can distinguish diabetic from obese diabetic patients.

**Conclusion:**

This study is the first to show a panel of serum miRNAs for obesity, and compare them with miRNAs identified in serum for diabetes and obesity with diabetes. Our results support the use of some miRNAs extracted from serum samples as potential predictive tools for obesity and type 2 diabetes.

## Introduction

Over the past decade, the prevalence of obesity in the world has dramatically increased across all age groups, especially in developed countries [1]. Obesity is characterized by abnormal or excessive fat accumulation that is the result of a chronic imbalance between energy intake and energy expenditure [2,3]. It poses a substantial health risk, as obesity is linked to several common diseases, such as type 2 diabetes (DM2), cardiovascular disease, stroke, arthritis, and several types of cancer [4]. Type 2 diabetes is one of the most prevalent metabolic disorders. DM2 is characterized by increased systemic glucose levels and insulin resistance. Many factors are contributing to the growing obesity and DM2 but genetic factors are thought to have great significance in their development. The investigation of gene expression regulatory mechanisms during the evolution of obesity and DM2 will have potential applications in prevention, early diagnosis and treatment. Micro-RNAs (miRNAs) are small, non-coding, 21-23 nucleotide long RNAs that negatively regulate gene expression by pairing with the 3’-untranslated region (UTR) of their target mRNAs [5]. miRNAs are involved in highly regulated processes such as proliferation, differentiation, apoptosis and metabolic processes. Several studies have highlighted the significance of miRNAs in maintaining metabolic homeostasis, and thus regulation of these miRNAs could serve as potential therapeutics in metabolic disorders [6,7]. MicroRNAs have been found in tissues and also in serum and plasma, and other body fluids, in a stable form that is protected from endogenous RNase activity. These unique characteristics of circulating miRNAs may provide a useful biomarker for supplemental diagnosis. Studies by Zampetaki et al [8] showed decreased levels of 10 miRNAs in plasma of diabetic patients (miR-15a, miR-20b, miR-21, miR-24, miR-126, miR-191, miR-197, miR-223, miR-320 and miR-486). The authors suggest that the five most significant regulated miRNA are both necessary and sufficient to distinguish DM2 patients (70%) from control (92%). This study also revealed that a decrease in circulating miR-126 expression is associated with the risk for future development of diabetes. In serum samples of recently diagnosed DM2 patients compared to DM2-susceptible subjects with normal glucose tolerance, Kong L et al. [9] found seven miRNAs (miR-9, miR-29a, miR-30d, miR-34a, miR-124a, miR-146a and miR-375) which were shown to be elevated. All these miRNAs have been previously related to insulin regulation [10]. However few studies have investigated circulating miRNA expression as potential biomarkers for obesity. Recently, Ortega FJ et al. [11] have showed deregulated expression of plasma miRNAs in morbidly obese men. They suggest that five miRNas (miR-142-3p, miR-140-5p, miR-15a, miR-520c-3c and miR-423-5p) may be novel biomarkers for risk estimation and classification of morbidly obese patients. Other papers have studied adipocyte-specific mRNAs and miRNAs that have also been detected in exosomes and microvesicles isolated from rat serum [12–14].

The aim of this profiling study was to characterize the expression of miRNA in serum samples of obese, nonobese diabetic and obese diabetic individuals. We wanted to determine whether miRNA expression was deregulated in these serum samples and to identify whether any observed deregulation was specific to either obesity or diabetes or obesity with diabetes. This study is the first to show a panel serum miRNA for obesity, and compare them with miRNAs indentified in serum for diabetes and obesity with diabetes.

## Materials and Methods

### Study population, blood collection and preparation of serum for miRNA studies

This project was approved by the Ethics Committee of Clinic San Carlos Hospital and written informed consent was obtained from all volunteers. A total of 69 individuals were categorized accordingly into four groups: 1) healthy controls (CTR) (n=20; 50%Females, 50% Males ), 2) type 2 diabetes (T2D) (n=13; 46%Females, 53% Males), 3) Obese (Ob) (n=20; 85%Females, 15% Males ) and 4) obese with type 2 diabetes (Ob-T2D) (n=16; 40%Females, 60% Males) based on guidelines of the World Health Organization and International Diabetes Federation [1] (Table 1). Diabetes was considered to be present if the individual had a fasting glucose ≥ 126 mg/dl, or a pre-established diagnosis of diabetes. Individuals with BMI ≥ 30 kg/m^2^ were classified as obese. Blood was collected in serum Vacutainer tubes with clot activator (Cat. 368968; Becton Dickinson) from volunteer donors at least 12 h following their most recent meal. Serum glucose was obtained by a glucose-oxidase method adapted to autoanalyzer (Hitachi 740, Boehringer Mannheim, Germany). Serum total cholesterol, trygliceride and high-density lipoprotein levels were measured using commercial kits (Boehringer Mannheim, Germany). Low-density lipoprotein was calculated by the Friedewald formula. For miRNA studies, one 10-mL serum tube with clot activator was collected. After allowing the content of serum tube to clot at room temperature for 1 h, serum was prepared by centrifugation at 1000 × g (2,300 rpm) for 15 min at 4°C in a KUBOTA 5900 centrifuge. The serum supernatant was slowly removed by using a plastic transfer pipette, leaving 0.5cm remaining to avoid disturbing the serum–clot interface, and stored at -80°C before use and were thawed on ice before use. 

**Table 1:** Clinical characteristics.

	**Controls**	**DM2**	**OB**	**OB-DM2**
**No. Subjects M/F %**	20; 50/50	13; 53/46	20; 15/85	16; 60/40
**Age (years)**	42.9±12.13	69.40±7.12*	41.7±11.18	67.55±11*
**BMI (Kg/m^2^)**	22.7±2.43	24.86±1.49	42.73±4.67*	33.38±2.86*
**Waist Perimeter (cm)**	79.45±7.77	91.53±6.75	110.56±10.26*	121.13±7.26*
**Fasting Glucose(mg/dl)**	91.27±7.25	126.57±23.09*	93.32±6.25	148.45±60.16*
**Insulin**	10.14±5.56	21.25±12.65*	29.24±13.2*	29.78±20.93*
**HOMA**	2.38±1.46	6.89±5.81	6.22±2.99	10.64±8.66
**Cholesterol (mg/dl)**	185.8±31.88	183.47±38.38	197.33±35.52	180.79±37.2
**LDL-C (mg/dl)**	108.77±24.59	84.22±26.97*	118.13±29.95	71.86±33.49*
**HDL-C (mg/dl)**	61.85±17.5	55.60±10.9	49.13±11.65*	50.16±7.87*
**SBP (mmHg)**	111.8±12.63	131±17	137±14	131±16
**DBP (mmHg)**	71.5±10.01	75±10	77±9.6	83±12
**Waist Circunferences (cm)**	79.45±7.77	91.53±6.75	110.56±10.26*	121.13±7.26*

Data are mean ± standard deviation. *p<0.05 when compared with control group. DM2, type 2 diabetes mellitus; OB, obesity; OB-DM2, type 2 diabetes mellitus and obesity; BMI, body mass index; LDL-C, low density lipoprotein cholesterol; HDL-C, high density lipoprotein cholesterol; SBP, systolic blood pressure; DBP, diastolic blood pressure.

### miRNA isolation and quality control of RNA

Total RNA was extracted from serum using a commercial column-based system following the manufacturer’s instructions with the following modifications (Qiagen miRNeasy Mini Kit). Serum was thawed on ice and centrifuged at 3000 x g for 5 min at 4°C in microcentrifuge. An aliquot of 200 µl of serum per sample was transferred to a new microcentrifuge tube and 750 µl of a Qiazol mixture containing 1.25 µg/mL of MS2 bacteriophage RNA (Roche Applied Science) and spike-ins were added to the sample. A rinse step (500 µL Qiagen RPE buffer) was repeated 2X. Total RNA was eluted by adding 50 µL of DNAsa-RNase-free water to the membrane of the spin column and incubating for 1 min before centrifugation at 15,000xg for 1 min at room temperature. The RNA was stored at -80 °C. To asses the quality of RNA isolated from the cell-free serum, endogenous microRNA assays were carried. For testing the purification yield and absence of PCR inhibitors, miR-191 and miR-423-3p (miRNAs typically detected in serum) were tested on each of RNA from 200 μl of serum from the individual patients/controls (Table S1). The samples must be detected with a Cp < 37 to be included in the analysis. Samples that did not pass this criteria were omitted from any further analysis.To identify hemolyzed samples, the level of miR-451 (microRNA highly abundant in RBCs) was assessed in RNA samples. In the hemolysed samples the concentration of miR-451 was significantly higher (Table S2), as compared with non-hemolysed samples. The hemolysed samples were removed from research.

### Reverse transcription and pre-screening

Two μl of RNA eluate was reverse transcribed in 10 μl reactions using the miRCURY LNA™ Universal RT cDNA synthesis kit (Exiqon). For the initial screening, the Human panel I and panel II containing 742 miRNAs (Exiqon) were applied to 4 pooled samples, one per group. Four ul of cDNA diluted 50 x (equivalent to 0.064 μl original serum sample), was assayed in 10 ul PCR reactions according to the protocol for mirCURY LNA™ Universal RT miRNA PCR. A no-template control (NTC) of water was purified with the samples and profiled like the samples. All amplifications were performed in a 7900HT Fast Real-Time PCR System (Applied Biosystems Inc.) in 384 well plates. The amplification curves were analyzed using the AppliedSDS2.4 software, both for determination of C_T_ (by the second derivate method) and for melting curves analysis.

### Pre-screening data analysis

All assays were inspected for distinct melting curves and the Tm was checked to be within known specifications for each particular assay. Furthermore any sample assay data point must be detected with 3 Cts less than the corresponding negative control assay data point, and with a Ct < 37 to be included in the data analysis. Data that did not pass these criteria were omitted from any further analysis. The default PCR procedure was used, and the analysis was performed by using RQ manager software (Applied Biosystems Inc.). ΔC_T_ and ΔΔC_T_ were calculated using the following mathematical formula: ΔC_T_=C_T sample_ - C_T Endogenous_, ΔΔCT= ΔC_T case_ - ΔC_T control_. We performed ΔC_T_ normalization, using GeNorm methodology. The GeNorm analysis suggested using the 4 most stable (rank-invariant) miRNAs (miR-, miR-30c, miR-103, miR-191 and miR-423-3p). The geometric mean of all the selected internal controls was used as a normalizing factor. Differential expression was calculated using the 2^−ΔΔCt^ method, and miRNAs were considered differentially expressed beyond a threshold of a 5 fold change. Our aim was to identify potential biomarkers for obesity or diabetes type 2. Because of this, for validation by RT-PCR, we selected miRNAs differentially expressed just in one patient group respect other groups.

### Analysis of individual miRNAs by Real-time quantitative PCR

We used miRCURY LNA™ microRNA PCR System (Exiqon) to assess the presence in serum of individual miRNAs. The cDNA was used as a template for the qPCR reaction using miRNA specific LNA™ PCR primer and Universal PCR primer. Gene expression levels were quantified using the 7900HT Fast Real-Time PCR System (Applied Biosystems Inc.) in 384-well plates. For the analysis by qRT-PCR in each extended sample, we first evaluated a suitable number of reference miRNAs, on the basis of increased expression stability and using the GeNorm methodology. The GeNorm analysis suggested including 4 reference miRNAs (miR-30C, miR-103, miR-191 and miR-423-3p). The selection and addition of various endogenous controls (reference miRNAs) for measures by qRT-PCR and the use of this geometric mean have been identified as among the most accurate and robust factors for normalization [15]. Thus, the geometric mean of all the selected internal controls was used as a normalizing factor. Relative expression was calculated using the comparative Ct method.

### Statistical analysis

Statistical analysis was performed with SPSS software version **15.0** (SPSS, Inc., Chicago, USA). A *P*-value <0.05 was considered statistically significant. For each miRNA, a receiver operating characteristic (ROC) curve was generated. The area under curve (AUC) value and 95% confidence intervals (CI) were calculated to determine the specificity and sensitivity. To increase the diagnostic accuracy of combined changes in serum miRNA levels, multiple logistic regression analysis was carried out according to previously described methods [16].

## Results

### Identification of potential miRNAs biomarkers in serum samples

To identify miRNAs in serum from diabetic and obese patients, which could act as a robust and reliable biomarker for these diseases we chose to use Exiqon QPCR panels .To detect the miRNAs most likely to be deregulated, we chose to pool samples within patients and controls. This pooling method had the advantage that we would only detect miRNAs that were expressed in the majority of subjects within the group. Therefore, this procedure reduced variation between individuals and enriched for miRNAs most likely to change between different groups. The four groups of serum samples comprised normal, healthy controls (CTR; *n*=20), diabetic patients (DM2; *n*=13), obese patients (OB; *n*=20) and diabetic with obesity (OB-DM2; *n*=16). Of the 739 miRNAs profiled by the Human panel I+II v2.M, (Exiqon), 244 miRNAs were detected in all serum pools with Ct values <35 (Figure S1; Table S3). This is the first report where it is observed a differential miRNA expression in patients with OB, DM2 and OB-DM2 and compared with healthy individuals and among themselves. The more different miRNA expression patterns were in OB compared with CTR (Correlation Pearson´s coefficient 0.392), OB-DM2 compared with CTR (Correlation Pearson´s coefficient 0.5417) and OB compared with DM2 (Correlation Pearson´s coefficient 0.696). Interestingly, the differences in miRNA expression pattern in OB-DM2 compared with DM2 or OB were almost similar (Correlation Pearson´s coefficient 0.822 and 0.8818 respectively). Therefore, OB showed a miRNA expression pattern different than the others groups. After the relevant quality control steps (see Materials and Methods), twelve miRNAs were selected. These selected miRNAs were detected in all patient/control groups with the expression pattern of each miRNA being different for at least one patient group. These twelve potential miRNA biomarkers, were chosen for further investigation; miR-101, miR-138, miR-15b, miR-150, miR-25, miR-205, miR-27b, miR-376a, miR-432-5p, miR-500a, miR-503 and miR-942. These miRNAs were selected because they showed different expression level between at least one patient group and other groups. We considered a miRNA differentially expressed when value of 2^−ΔΔCt^ was at least 5. 

### Validation by RT-qPCR in individual serum samples

**Figure 1:**
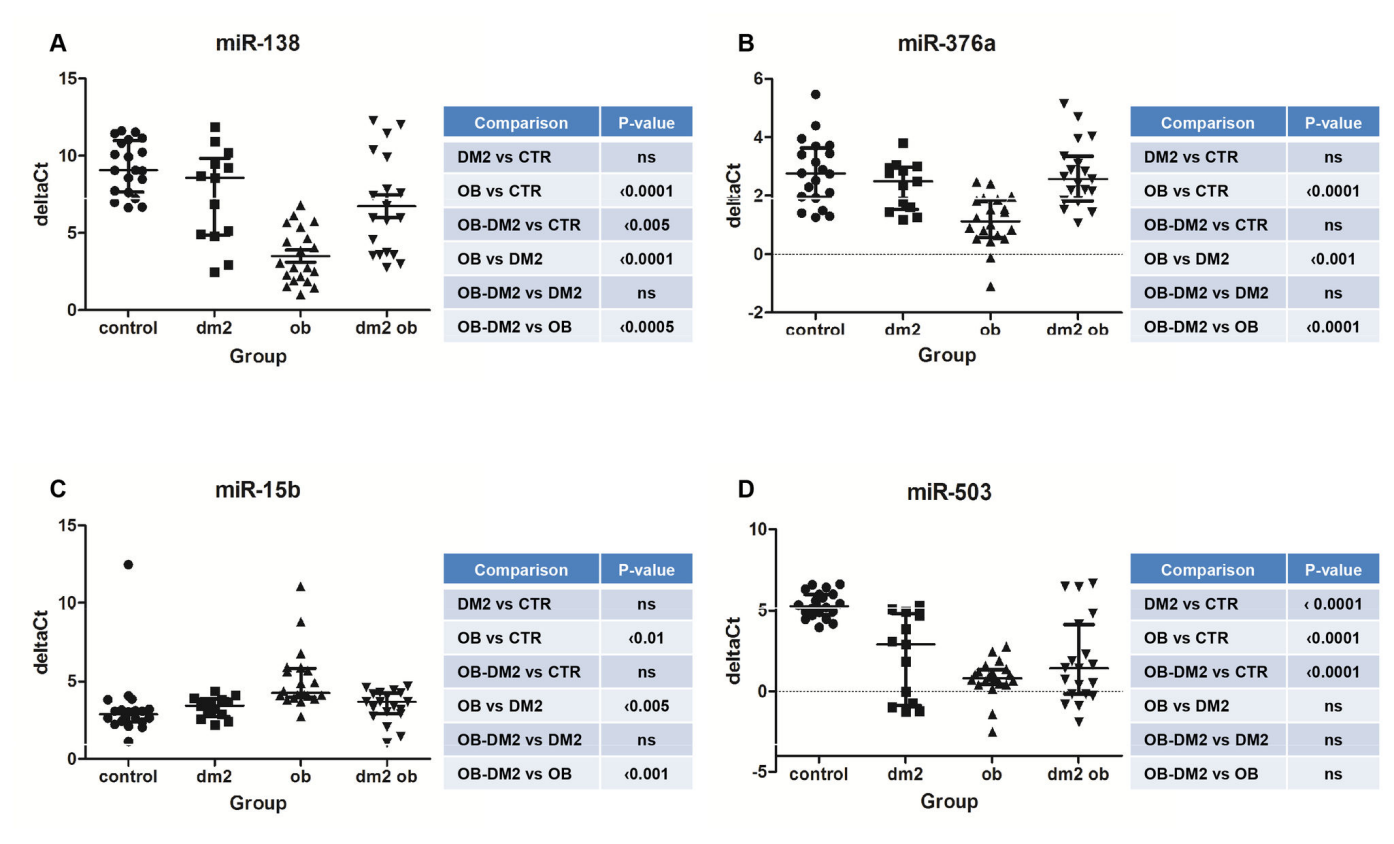
Comparison of the serum levels of miR-138(A), miR-376a (B), miR-15b (C) and miR-503 (D) in controls, DM2, OB and OB-DM2 groups. Graphs show median level with interquartile range. The Kruskal-Wallis test with Dunn´s post test was used to determine the significance of differences between groups. The serum levels were determined using RT-PCR following RNA extraction. Two technical replicates were performed on two cDNA replicates.

To validate the potential biomarkers identified from the prescreening results, serum levels of these miRNAs were measured by qRT-PCR assays. As described previously in *Materials and Methods*, the geometric mean of all the selected internal controls (miR-30C, miR-103, miR-191 and miR-423-3p) was used as a normalization control in serum. Serum miR-30C, miR-103, miR-191 and miR-423-3p levels were evaluated in all subjects (patients and controls). Our data demonstrated that no significant difference was observed in term of Ct values of these miRNAs between control and patient samples (Table S4). Serum levels of the 12 selected miRNAs were validated by qRT-PCR on the 69 serum samples (Figure S2; Table S5). Our data indicated that all the 12 miRNAs were detected in serum but only miR-138 and miR-376a in obese serum were significantly diminished and serum miR-15b level significantly higher when compared to controls, diabetic and obese diabetic (all *p-values*<0.01) (Figure 1A,1B, 1C respectively). Therefore, these three miRNAs could be potential biomarkers of obesity. None of the validated miRNAs were useful as biomarkers of diabetes type 2. Only miR-503 in diabetic serum was significantly lower when compare to controls (Figure 1D). But in serum samples from obese patients, miR-503 also was significantly lower when compared to controls (Figure 1D). Therefore miR-503 alone could not be used as a biomarker of diabetes type 2.

### Evaluation of the biomarker potential of miR-138, miR-376a and miR-15b to distinguish OB patients from normal healthy control

**Figure 2:**
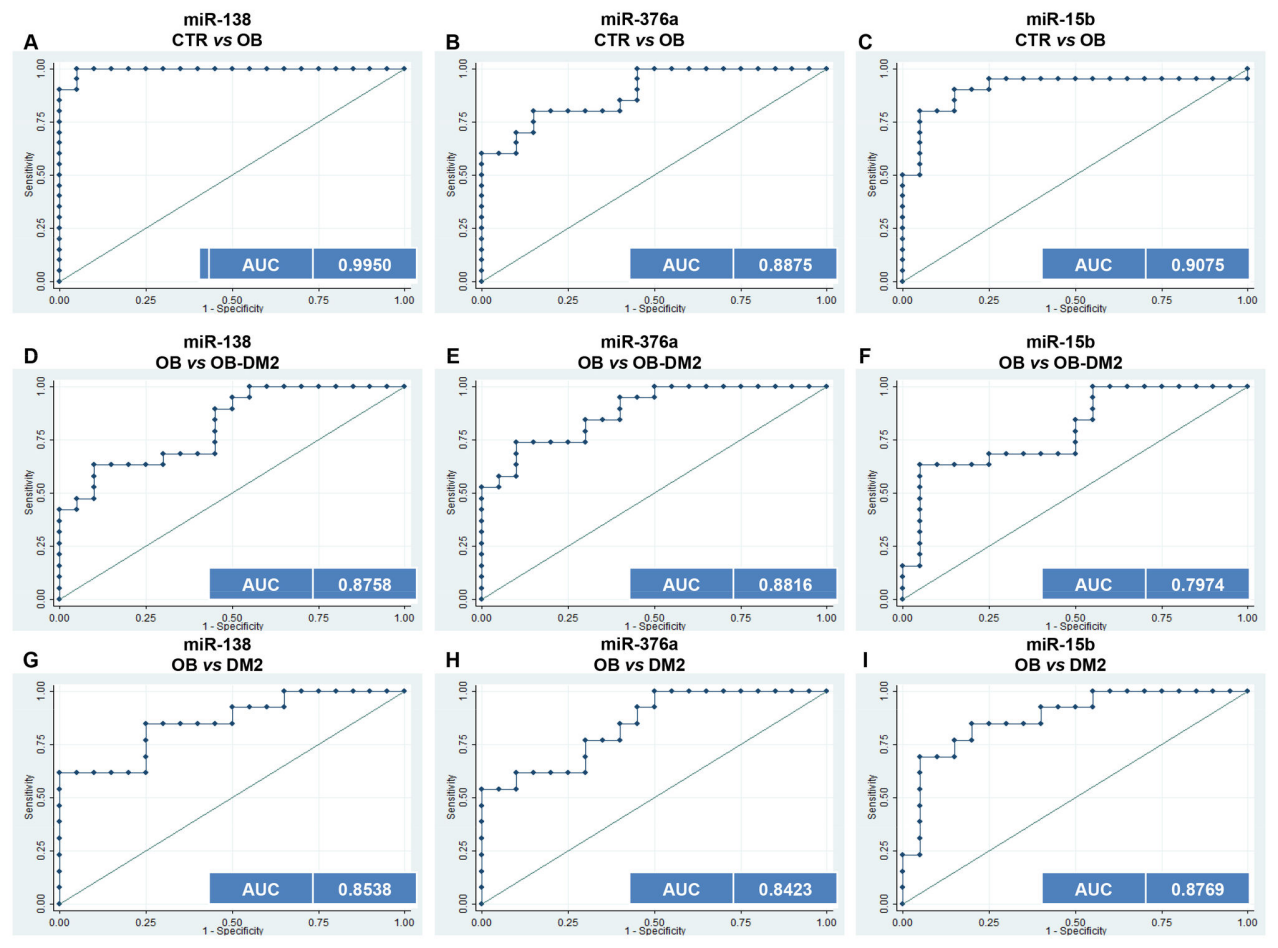
ROC curves to compare the ability of each miRNA to distinguish between different groups. (**A**) miR-138, (**B**) miR-376a and (**C**) miR-15b can distinguish between OB and CTR. (**D**) miR-138 (**E**) miR-376a (**F**) miR-15b can also distinguish between OB and OB-DM2. (**G**) miR-138 (**H**) miR-376a (**I**) miR-15b can distinguish between OB and DM2.

Receiver operating characteristic (ROC) curve analyses showed that the ROC curve areas for miR-138, miR-376a and miR-15b alone were 0.9950 (95% CI, 0.98310-1.0000), 0.8875 (95% CI, 0.78905-0.98595) and 0.9075 (95% CI, 0.6543-0.9404) respectively (Figure 2A, 2B, 2C). Although miR-15b and miR-376a alone generated satisfactory ROC values, miR-138 (Figure 2A) showed ROC values for discriminating OB patients from healthy control. Therefore, use of miR-138 alone provides excellent discrimination between OB patients and healthy subjects. 

### Evaluation of the biomarker potential of miR-138, miR-376a and miR-15b to distinguish OB patients from OB-DM2 and DM2 patients

To determine whether the levels of serum miRNAs can be used to distinguish obese patients from OB-DM2 and DM2 patients, we established ROC curves to analyze the difference in the levels of serum miR-138, miR-376a and miR-15b between groups. Comparing OB subjects with OB-DM2, ROC curve areas of miR-138, miR-376a and miR-15b were found to be, 0.8758 (95% CI: 0.6838-0.9477), 0.8816 (95% CI, 0.7797-0.9835) and 0.7974 (95% CI, 0.6543-0.9404) respectively (Figure 2D, 2E, 2F). These results clearly showed that the levels of serum miR-138 and miR-376a, can distinguish patients with OB from OB-DM2. We next compared the levels of these serum miRNAs between OB and DM2. ROC curve areas of miR-138, miR-376a and miR-15b were, 0.8538 (95% CI: 0.7165-0.0.9912), 0.8423 (95% CI, 0.7065-0.9781) and 0.8769 (95% CI, 0.7546-0.9992) (Figure 2G, 2H, 2I). These results also demonstrated that the levels of these three miRNAs can distinguish patients with OB from DM2. Together, these results demonstrated that the levels of miR-15b, miR-138 and miR-376a in serum can be used to distinguish OB from OB-DM2, DM2 and healthy control.

### Evaluation of the biomarker potential of miR-503 and miR-138 in combination for distinguish DM2 from OB-DM2 patients

**Figure 3:**
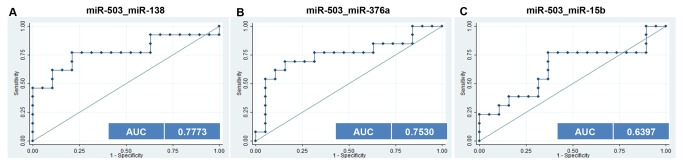
ROC curves to compare the ability of combinations of miRNAs to distinguish between DM2 and OB-DM2 groups. The combination of miR-503 and miR-138(**A**) and miR-503 and miR-376a (**B**) could distinguish DM2 from OB-DM2 patients. (**C**) The combination of miR-503 and miR-15b cannot distinguish between DM2 and OB-DM2 patients.

Our results show that single miRNA cannot distinguish between DM2 and OB-DM2 (Figure 1). miR-503 was only one that allows differentiation between DM2 or OB-DM2 from healthy controls (Figure 1D). In multiple logistic regression analysis of miR-503 and miR-138 the resulting ROC curve shows reasonable separation between the two groups (AUC=0.7773; CI 0.5886-0.9661) (Figure 3A). The multiple logistic regression analysis of miR-503 and miR-376a is also able to make the distinction (AUC=0.7530; CI 0.5596-0.9465) (Figure 3B). However, this analysis of miR-503 and miR-15b cannot distinguish these conditions (Figure 3C). It is important that the miRNA biomarkers can distinguish patients with obesity and diabetes from patients with obesity or healthy controls. Such a test would be useful to aid in the prediction of diabetes.

## Discussion

Circulating miRNAs have been extensively investigated as novel and non-invasive diagnostic and prognostic markers. Most published studies have been focused on different types of cancer [17–19]. More recently, the role of miRNAs in diabetes and obesity has started to be studied. In this study, we performed an initial pre-screening with Exiqon panels followed by qRT–PCR validation to screen human miRNAs for potential to act as biomarkers in diabetes and obesity. We identified three serum miRNAs, miR-138, miR-376a and miR-15b whose concentrations were significantly deregulated in the serum of OB patients compared with OB-DM2, DM2 and normal controls (Figure 1A, B, C). ROC curves, revealed that the three miRNA panel has a promising ability to distinguish OB individuals from OB-DM2, DM2 and normal healthy controls (Figure 2). Use of miR-138 and miR-503 in combination showed good ability to efficiently distinguish DM2 from OB-DM2 patients (Figure 3A). This combination of miRNAs can also be used to distinguish DM2 and OB-DM2 patients from normal controls. Therefore, these miRNAs show great potential as predictive test, which can be used both in the clinic and for screening the general population. We have also shown that miR-376a and miR-503 together can distinguish DM2 from OB-DM2 patients (Figures 3B). This is the first time that serum miRNAs have been shown to act as useful predictive biomarkers in obesity and obesity-related diabetes. Moreover, these miRNAs that we have found to be deregulated in obese patients have not yet been found in the circulating blood in association with this metabolic condition.

Deregulation of miRNAs is known to be involved in multiple processes including cell proliferation, apoptosis, cell-cycle regulation inflammation and invasion in various diseases. Among the three serum miRNAs identified in this study, some have already been reported to play important roles in obesity or diabetes. For example, miR-138 is down-regulated during adipogenic differentiation in human multi-potent MSCs [20]. miR-138 has been demonstrated to target the 3’UTR of EID-1, an interacting inhibitor of differentiation that can interact with SHP, an endogenous enhancer of adipogenic PPARγ2 [21].Therefore miR-138 appears to indirectly regulate PPARγ, an established transcription factor driving adipogenic gene expression in human MSCs [22]. The up-regulation of miR-15b is observed in the regenerating mouse pancreas as compared to embryonic day (e) 10.5 or e 16.5 developing mouse pancreas [23], which is associated with regulation of Nerogenin3 (NGN3), a bHLH transcription factor, marks pancreatic endocrine progenitor cells, as confirmed by lineage tracing studies [24] and is essential for expression of insulin in mouse liver cells, acinar cells, gut cells and ES cells [25,26]. *ngn3* mutant mice develop diabetes and die at early postnatal stages [27]. Perhaps, deregulating of miR-15b in serum obese patients that we observed in our results could be implicated in a later development of type 2 diabetes. Prospective study would be necessary to validate this hypothesis. Additionally, Zhang Y et al [28], showed that the expression of miR-15b was also significantly elevated in the serum of fatty liver disease patients compared with healthy subjects. Non-alcoholic fatty liver disease (NAFLD) is a type of liver disease induced by long-term excessive energy intake, and it is strongly associated with type 2 diabetes, obesity and hyperlipidemia [29,30]. Up-regulation of miR-15b was also observed in the high-fat-induced non-alcoholic fatty liver disease (NAFLD) SD rat model and in the palmitate-induced NAFLD L02 cell model [28]. Increased mir-15b expression in NAFLD models may lead to decreased cell proliferation and glucose consumption while inducing the storage of intracellular triglyceride, which are all hazards of NAFLD and obesity.

However, there are no reports about the role of miR-376a in obesity. In hepatocellular carcinoma cells HCC, miR-376a is significantly down-regulated and the elevated miRNA-376a repressed cell proliferation and induced apoptosis in HCC cells by targeting p85α and reduced PIK3R1 directly [31]. Apoptosis is a fundamental mechanism for maintaining homeostasis by removing dangerous and unnecessary cells. However, adipocytes are resistant to apoptosis because of high levels of Akt/protein kinase B and the anti-apoptotic factor Bcl-2. Adipocytes could be removed through apoptotic mechanisms in some pathological conditions such as obesity. The induction of apoptosis in adipocytes, by regulating miR-376a could be a possible method to reduce the adipocyte number. 

Therefore, these three miRNA identified in this study could be implicated in adipogenesis, pancreatic regeneration, proliferation and apoptosis. All these processes are important in an environment of obesity, but our results show that these miRNA present significant differences between OB and healthy control and what is more important, these miRNAs allow differentiating between OB and OB-DM2 patients. Progression to overt diabetes in obese subjects is not always predictable. Thus, while some obese individuals progress to type 2 diabetes, others may only have mild metabolic abnormalities, suggesting that the absolute amount of fat stored may not be the most important factor determining the relationship between obesity and type 2 diabetes [32]. Indeed, other factors such as adipose tissue inflammation are viewed as key promoters of progression to type 2 diabetes [33]. Recently, miR-138 was reported to be down-regulated in esophageal squamous cell carcinoma (ESCC) and the markers of lipid rafts FLOT1, FLOT2 and caveolin-1 were identified as its targets, and NF-kappaB was activated [34]. Future studies will be necessary to explore if the down-regulation of miR-138 in serum samples of obese patients may also play an important role in inflammation process of the obesity.

In this study, a serum 3-miRNA-based expression profile that was able to accurately discern OB individuals from normal controls and OB-DM2 and DM2 patients had been identified. However, some of the highly expressed miRNAs were different from those found in previous studies. This inconsistency may be mainly due to differences in miRNA sources or to the difference between intracellular miRNAs and extracellular miRNAs. Other factors, such as study design, race, sample size or methodology may have also influenced the final results. These findings may have implications in the understanding of OB, establishing management strategies and estimating prognosis.

An important question arises about the potential impact of the pharmacological treatments used in diabetes, obesity and associated conditions on any of the identified miRNAs. Virtually no studies have addressed this issue in clinical setting. However, only Zampetaki et al. [8] showed similar levels of 13 miRNAs in plasma (miR-15a, miR-20b, miR-21, miR-24, miR-126, miR-191, miR-197, miR-223, miR-28-3p, miR-150, miR-29b, miR-320 and miR-486) of diabetic patients with or without drug treatment, mainly sulfonylureas. In fact, studies evaluating the role of drugs on miRNA regulation in metabolic disorders are recommended.

In conclusion: This study is the first to show a panel of serum miRNAs for obesity and compare them with miRNAs identified in serum for diabetes and obesity with diabetes. Moreover, our study goes to support the use of miR-15b, miR-138 and miR-376a extracted from serum samples as potential predictive tools for obesity and type 2 diabetes.

## Supporting Information

Figure S1 **Heatmap after removing all miRNAs with Ct values > 35.**
(TIF)

Figure S2 **Heatmap miRNAs validated by RT- quantitative PCR.**
(TIF)

Table S1 **Raw data of quality control of the RNA isolated from serum samples.**
(XLSX)

Table S2 **Raw data of hemolysis control.**
(XLSX)

Table S3 **Raw data of the initial sreen.**
(XLSX)

Table S4 **Raw data endogenous controls.**
(XLSX)

Table S5 **Raw data of individual miRNAs validated by RT-PCR.**
(XLSX)
