# Supplementary figures and images for: Serum Circulating microRNA Profiling for Identification of Potential Type 2 Diabetes and Obesity Biomarkers
Source: PLoS One. 2013 Oct 15;8(10):e77251. doi: 10.1371/journal.pone.0077251 (PMC3817315; doi:10.1371/journal.pone.0077251)

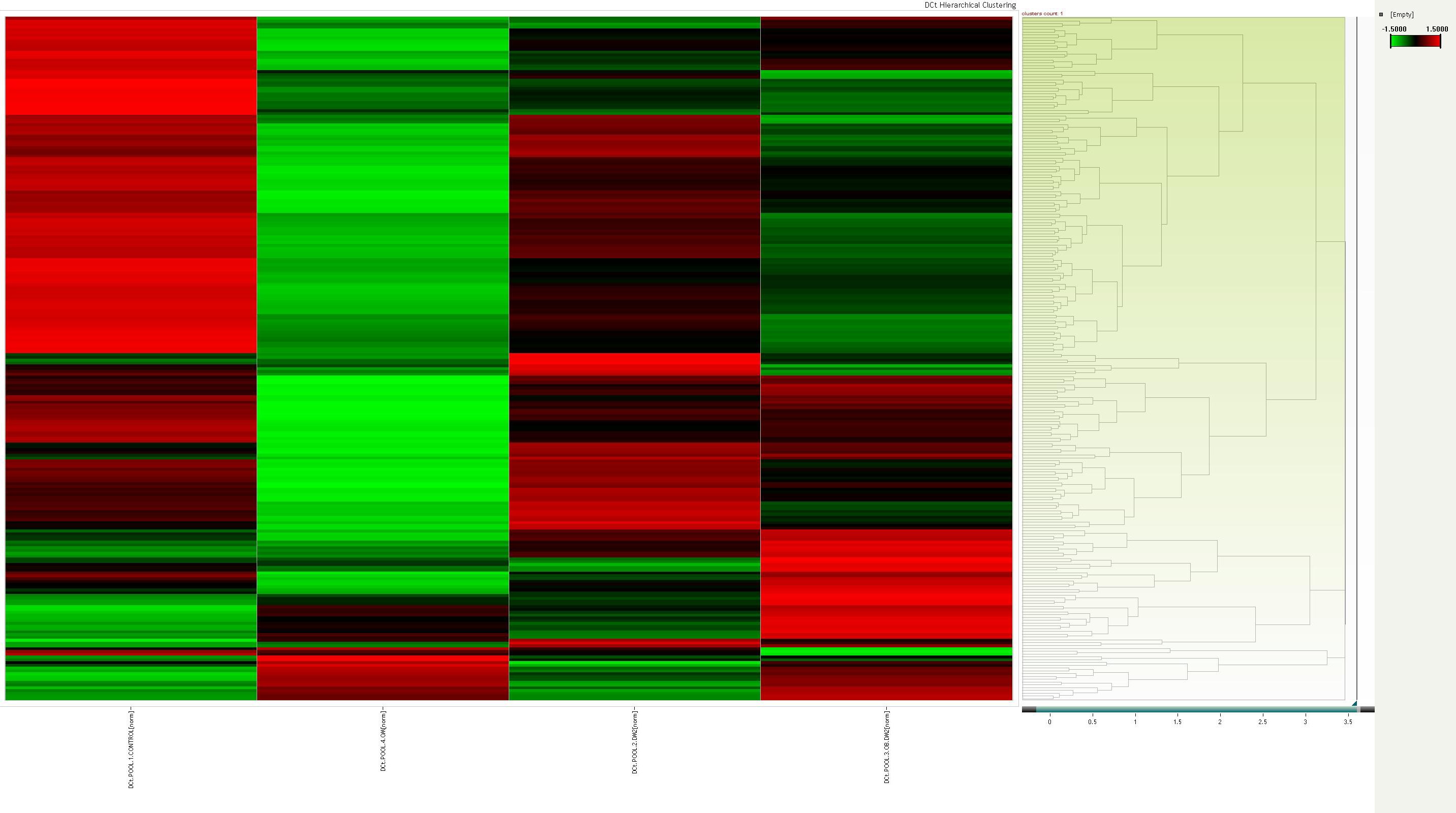

Supplement: Figure S1 — Heatmap after removing all miRNAs with Ct values > 35. (TIF) [file pone.0077251.s001.tif]

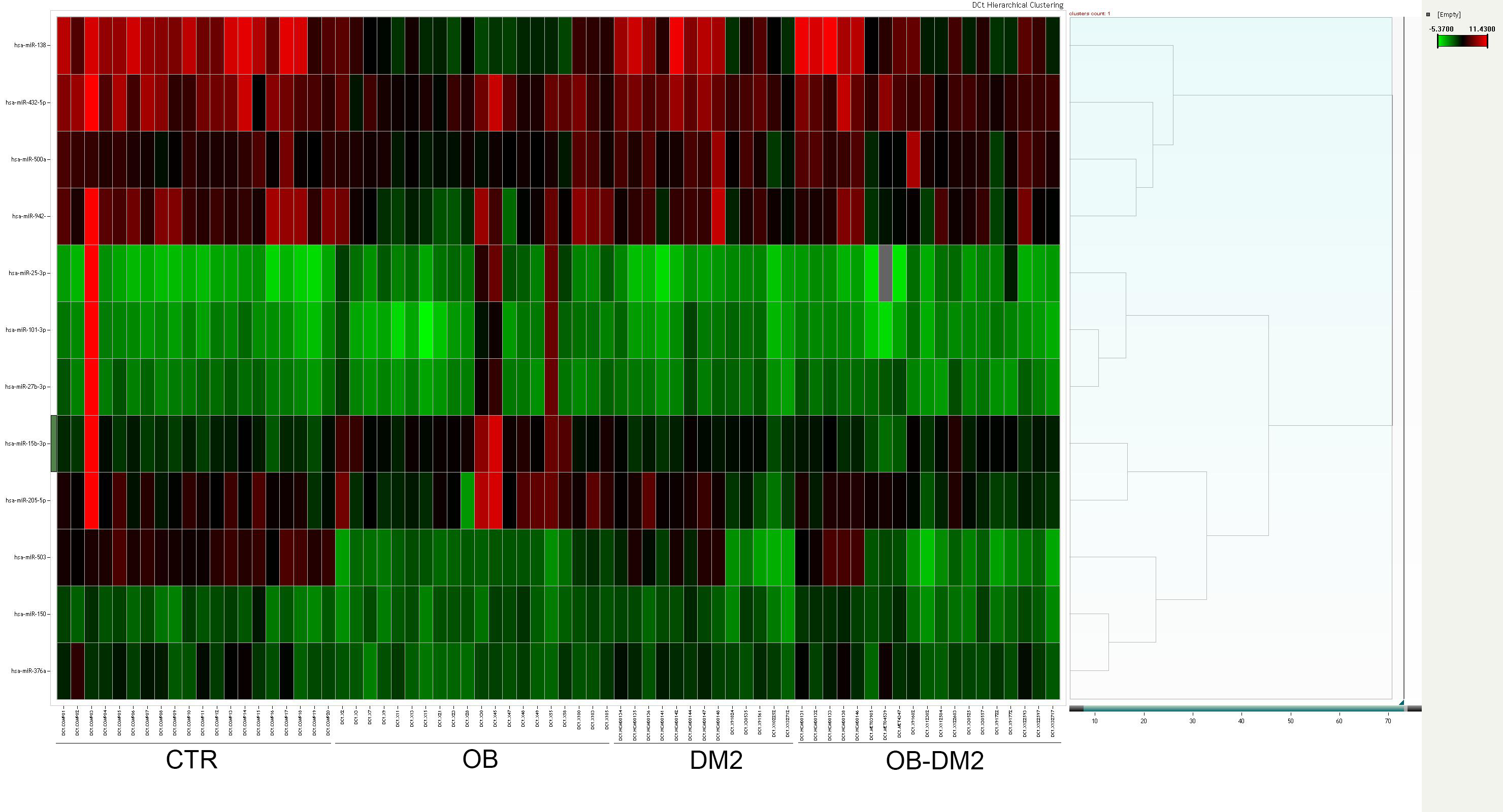

Supplement: Figure S2 — Heatmap miRNAs validated by RT- quantitative PCR. (TIF) [file pone.0077251.s002.tif]
